# From Personalization to Therapeutic Continuity: Framework for Memory in AI-Powered Mental Health Systems

**DOI:** 10.2196/99950

**Published:** 2026-08-28

**Authors:** Courtney Jewell, Kelsey McAlister, Tara Deliberto, Tanner Wallis, Grant Winns, Jennifer Huberty

**Affiliations:** 1 Fit Minded, Inc, 2901 E Greenway Rd PO Box 30271, Phoenix, AZ, 85046, United States, 1 (602) 935-6986; 2 Yuna Health, San Francisco, CA, United States

**Keywords:** artificial intelligence, AI safety, mental health, memory, therapeutic continuity, conversational agents, digital mental health intervention, longitudinal care, personalization, case formulation

## Abstract

AI-powered mental health tools are increasingly deployed to support users across multiple sessions, yet the field lacks a principled framework for how memory in these systems should be structured and applied. In most current implementations, memory functions primarily as a personalization mechanism, optimizing for conversational continuity and user engagement without distinguishing between types of information that have fundamentally different clinical relevance. We propose a framework organizing memory in AI-powered mental health systems into 4 functionally distinct types. Episodic memory captures discrete, time-bound experiences tied to specific events and context. Pattern memory, adapted from the concept of procedural memory in cognitive psychology, tracks recurring patterns in cognition, emotion, and behavior across sessions. Semantic memory captures stable, personally relevant background context about the user. State-responsive memory represents the user’s current emotional and psychological condition in real time, taking priority over the other 3 types when acute distress or risk is signaled. Each type corresponds to a distinct therapeutically relevant function, and together they are designed to support the kind of cumulative, longitudinal understanding that effective mental health care requires. We term this framework “therapeutically informed memory,” drawing on established memory systems research and applying it to the clinical requirements of AI-powered mental health support. The aim of this viewpoint paper is to give AI developers, clinicians, and mental health organizations a shared vocabulary and design framework for organizing memory around therapeutic function rather than personalization alone. This paper is intended primarily for AI product and engineering teams, clinical advisors to digital mental health companies, and researchers evaluating AI-powered mental health tools. We describe the design requirements and clinical rationale for each memory type, discuss how the types interact and how priority should be assigned across them, and use Yuna, an AI-powered digital mental health intervention developed with clinical input, as an illustrative example of how this framework can be applied in practice. We conclude with design implications for the field and identify open questions regarding memory quality metrics, outcome validation, and the ethical dimensions of persistent memory as priorities for future research.

## Introduction

AI-powered mental health tools are increasingly designed to support users across multiple sessions rather than within a single, isolated interaction [1,2]. This shift toward longitudinal engagement has prompted growing interest in how these systems manage and use information over time, a function commonly referred to as memory. In most current implementations, memory is treated primarily as a mechanism for personalization: the system recalls past topics, noted preferences, and conversational history in order to make interactions feel more coherent and tailored to the individual [3,4]. The goal, broadly, is to improve the user experience and sustain engagement. Throughout this paper, the term “AI-powered mental health system” is used broadly to encompass wellness and coaching chatbots, workplace mental health tools, digital therapeutics, clinician-supported platforms, and crisis-support tools. The applicability of the proposed framework and the rigor required to implement it responsibly vary across this range.

This framing reflects priorities that are well suited to consumer technology but are insufficient for mental health support. In clinical care, memory serves a fundamentally different purpose, one oriented toward therapeutic continuity: the capacity of a care system to build cumulative understanding of a person across time, tracking how their condition is changing and using that understanding to guide responses appropriately. Clinicians do not remember prior sessions primarily to create continuity of tone. They use longitudinal information to track changes in symptom severity, recognize patterns in cognition, emotion, and behavior, and make informed decisions about when and how to intervene [5]. Memory, in this sense, is a clinical function. It is what allows care to build across sessions rather than restart with each encounter, and it is central to identifying risk, recognizing deterioration, and supporting meaningful progress over time [6,7].

The distinction matters because conflating personalization with therapeutic continuity creates a specific category of design failure. A system that remembers what a user enjoys discussing is not the same as a system that tracks whether a recurring pattern of thinking has intensified, stabilized, or shifted over time. Without this distinction, AI mental health tools risk missing signals that a human clinician would recognize as clinically significant. Personalization and engagement, the dimensions most current systems optimize for [8,9], are insufficient for this purpose because they are measures of how an interaction feels to the user rather than measures of whether the system’s understanding of the user’s clinical trajectory is accurate. Neither dimension requires the system to detect change over time, weigh contradictory evidence, or distinguish a momentary state from an enduring trait [6,7]. This is needed to be able to recognize deterioration, nonresponse, or risk [6,7].

Despite rapid growth in the development and deployment of AI-powered mental health platforms [10,11], the field lacks a clear conceptual framework for how memory should be structured and applied in this context. Most systems do not distinguish between different types of memory or specify how each type should inform the system’s responses [12,13]. As a result, memory use tends to be either too broad, retrieving past context indiscriminately, or too shallow, failing to connect current experience to longitudinal patterns in meaningful ways.

The purpose of this paper is to address that gap. We propose a framework for therapeutically informed memory in AI mental health systems, organized around four functionally distinct memory types: episodic, pattern, semantic, and state-responsive. Each memory type captures a different kind of information and serves a different function in how the system understands and responds to the user over time. We describe the design requirements and clinical rationale for each memory type, discuss how they interact and how priority should be assigned across them, and use Yuna, an AI-powered digital mental health intervention developed with clinical input, as an illustrative example of how this framework can be applied in practice. We conclude with design implications for the field and identify open questions that warrant further investigation.

## Personalization Memory Versus Therapeutic Memory

Memory in AI systems can serve more than one purpose, and the distinction between those purposes matters considerably in a mental health context. In most current AI-powered mental health implementations, memory functions primarily as a personalization mechanism (ie, personalization memory). The system stores information gathered across interactions, including topics the user has discussed, preferred communication styles, and details the user has shared about their life, and draws on this information to make subsequent conversations feel more familiar and contextually relevant [3,14]. The goal of this approach is to create a sense of being understood and sustain engagement.

However, personalization memory is oriented toward the surface of experience. It captures what a user has talked about and how they prefer to be addressed but does not necessarily track what those conversations reveal about patterns of thinking, recurring emotional responses, or changes in symptom severity over time. The result is a form of continuity that may feel meaningful to the user without being clinically informative. A system can remember that a user often discusses work stress while having no structured representation of whether that stress has intensified over the past month, whether it is linked to a recurring cognitive pattern, or whether the user’s overall functioning has changed.

In clinical practice, memory serves a different and more specific function. Clinicians use longitudinal information to construct and continuously update a working model of the user’s presenting problems, including the factors that maintain those problems over time and the ways in which the user’s condition is changing [5,15]. This process, commonly formalized as case formulation, is not a static record of what the client has said [16]. It is an evolving, hypothesis-driven interpretation that is revised as new information accumulates across sessions, encompassing not only discrete events and self-reported experiences but also patterns that may not be immediately visible within any single interaction. Remembering more, in this sense, does not mean understanding more.

This distinction is reinforced by the evidence base for measurement-based care, which demonstrates that systematically tracking symptom change across sessions improves treatment outcomes, reduces dropout, and helps clinicians identify nonresponse before it becomes entrenched [6,7]. The clinical value of longitudinal information is not simply additive. It is qualitatively different from in-session observation because it captures trajectory rather than state, distinguishing a user who presents with moderate stress that is improving from one who presents with the same level of stress that has been stable or worsening for weeks [6]. Without this perspective, responses to the same expressed content may be functionally identical even when the underlying clinical picture is quite different.

The risks of treating memory primarily as a personalization tool in mental health contexts, therefore, go beyond a missed design opportunity. Without a structured model of what memory is for and how it should be organized, an AI system may reinforce unhelpful narratives rather than identify them, apply responses that were appropriate in one context to a situation that has materially changed, or fail to register accumulating signals of distress that would be apparent in any longitudinal clinical review. Personalization and engagement metrics are optimized for how an interaction feels in the moment and do not capture this kind of change. This is why strong performance on these metrics is consistent with missing a worsening trajectory entirely.

Ultimately, the distinction between personalization memory and what we term therapeutic memory is not a technical one. It is conceptual. Both can coexist within the same system, but they require different design logic and serve different purposes. Conflating them, or treating personalization as a proxy for therapeutic continuity, leaves a gap at the center of what AI mental health systems need to do well.

## A Framework for Therapeutically Informed Memory

The distinction between personalization and therapeutic memory points toward a practical design question: if memory in AI-powered mental health systems is to serve therapeutically relevant functions, how should it be organized? We propose that memory should be organized into 4 functionally distinct types, each corresponding to a different kind of information and a different role in how the system understands and responds to the user over time. Episodic memory captures discrete, time-bound experiences tied to specific events and context [17]. Pattern memory, adapted from the concept of procedural memory in cognitive psychology [18], tracks recurring patterns in cognition, emotion, and behavior across sessions. Semantic memory, drawn from the cognitive psychology concept of general world knowledge [17,19], captures stable, personally relevant background context about the user. State-responsive memory represents the user’s current emotional and psychological condition in real time, taking priority over the other 3 types when acute distress or risk is signaled [20]. Together, these 4 types form a framework we term therapeutically informed memory, drawing on established memory systems research and applied to the clinical requirements of AI-powered mental health support.

## Episodic Memory

Episodic memory, as defined in cognitive psychology, refers to the recollection of specific events tied to a particular time and context [17]. In an AI-powered mental health system, the equivalent function is the capture and organization of discrete, time-bound experiences reported by the user: a significant argument, a moment of acute distress, a realization during a session, and a period of poor sleep following a stressful week. These are the raw materials of narrative continuity. Without them, each session begins with no shared reference to what the user has actually lived through, and the system cannot situate current concerns within a meaningful personal history.

The design requirement for episodic memory is temporal organization. Events need to be stored in a way that preserves their sequence and context, not simply accumulated as a pool of undifferentiated past content. This allows the system to recognize when a current concern echoes a previous experience, to reference a relevant past event when appropriate, and to build a coherent timeline of the user’s experience rather than treating each session as independent. Selective retrieval is equally important: not all past events are relevant at a given moment, and surfacing irrelevant history can be as unhelpful as having none at all. Clinically, episodic memory corresponds to the session history and time-bound events that appear in a client’s chart, such as the discrete incidents a clinician references when tracking how a specific stressor or crisis has unfolded over time. For transparency and auditability, episodic memory is best represented as timestamped, structured event records, so the individual events can be reviewed, corrected, or removed without altering the raw conversational transcripts. Events should be timestamped with a contextual relevance score so that older, less relevant experiences are weighted appropriately rather than treated as equivalent to more recent events.

## Pattern Memory

In cognitive psychology, procedural memory refers to knowledge acquired through repetition that is expressed through behavior rather than conscious recall, encompassing motor skills, habits, and conditioned responses [18]. We adapt this concept for clinical application and term it pattern memory, reflecting its function in an AI-powered mental health context: the recognition and tracking of recurring patterns in cognition, emotion, and behavior across sessions. Like procedural memory, pattern memory is not built from single exposures. It emerges through accumulation, as repeated instances across time reveal an underlying structure that is not visible within any individual interaction [18]. This is an analogical adaptation rather than a direct clinical mapping. The underlying regularity is built through accumulation and expressed through repetition rather than recalled as a discrete event. That same property is what makes recurring cognitive, emotional, and behavioral patterns invisible within a single session but visible once instances are considered together across many. Pattern memory should be treated as an inferential layer rather than a simple memory store. Therefore, its outputs require uncertainty representation, validation, and ongoing clinical oversight.

In clinical practice, the analogous function is well established. A user who consistently catastrophizes in response to uncertainty, who withdraws socially during periods of low mood, or who interprets criticism as evidence of personal failure is exhibiting patterns that a clinician would track, name, and return to across sessions [21,22]. An AI system without pattern memory can respond appropriately to what a user says in a given session while remaining blind to what that session reveals when placed alongside others. The design requirement is aggregation and pattern detection across sessions, with mechanisms to update the system’s representation of a pattern as new instances confirm, qualify, or contradict it. Clinically, pattern memory corresponds to the maintaining factors, cognitive distortions, avoidance cycles, schema patterns [21,22], and relapse signatures that case formulation [5] is designed to identify and track. A pattern should move from where it is first observed, candidate status, to established status only after it recurs across multiple sessions and is supported by more than one source of evidence. Structured self-report and confirmation through in-session inquiry should be weighed more heavily than AI inference from conversational content alone. When a later session contradicts an established pattern, that instance should weaken or begin retiring the pattern rather than being discounted. This allows the system not to permanently label a user based on outdated information. Exact numeric thresholds for these transitions remain an empirical question outside the scope of this conceptual framework, and thus we treat them as parameters to be established and validated in future work. Pattern memory is best captured as a temporally organized graph structure connecting cognitive, emotional, and behavioral nodes across sessions so that its inferential status and supporting evidence remain visible and auditable.

## Semantic Memory

Semantic memory in cognitive psychology broadly refers to general world knowledge independent of personal experience [18] but is adapted here to capture stable, personally relevant background context about the user [19]: their relationships, living situation, employment context, significant life history, and other facts that remain relatively constant and provide the interpretive frame within which more dynamic information is understood. Knowing that a user is a primary caregiver for an ill family member, or that they recently experienced a significant loss, changes how the AI system should interpret expressions of exhaustion, withdrawal, or hopelessness.

Semantic memory requires structured, high-accuracy storage that is kept distinct from the more dynamic information captured in episodic and pattern memory. It is not updated session by session in the way that pattern recognition or event history is, but it requires maintenance when circumstances change and careful accuracy because errors in background context can systematically distort how other information is interpreted. Clinically, semantic memory corresponds to the stable psychosocial context, life circumstances, relationships, work environment, and risk or protective factors that inform case conceptualization [5] independent of moment-to-moment presentation. Semantic memory is best represented as discrete structured records because it functions as an interpretive frame for the other memory types. This allows each fact to be individually reviewable, correctable, and subject to periodic reconfirmation so that background context does not become irrelevant as a user’s life circumstances change.

## State-Responsive Memory

The fourth type addresses a dimension that the preceding three do not fully capture: the user’s current condition in real time. State-responsive memory is a representation of the user’s immediate emotional and psychological state, including intensity of distress, expressed hopelessness, signals of acute risk, and other features of the present moment that require timely and appropriate responses. Unlike the other memory types, which are fundamentally longitudinal, state-responsive memory is anchored in the current session and updated continuously as the interaction unfolds. This distinguishes it from trait-level responses, which reflect stable, cross-situational tendencies. Clinically, state-responsive memory corresponds to real-time risk assessment, crisis response [23,24], and the prioritization of acute distress and safety over longitudinal considerations. State-responsive memory is best represented as a session-scoped state rather than a persisted record because it is transient by design. If any element of it is retained beyond the session, that retention requires an explicit rationale and its own governance rather than being treated as an extension of episodic memory.

Its defining clinical feature is priority. When the user’s current state signals significant distress or risk, responses should be calibrated to the present moment rather than the trajectory, consistent with how crisis-informed clinical practice operates: longitudinal knowledge informs understanding, but immediate safety and stabilization take precedence [23,24]. The design requirement is real-time updating with clear prioritization rules that govern when and how state-responsive memory overrides or modifies the application of the other 3 types. The priority should be graded rather than binary. Override is triggered by explicit signals, such as expressed hopelessness, expressed intent, or other validated acute risk indicators [25,26], and its strength should scale with the specificity and severity of those signals. This matters because state-responsive memory should not categorically suppress the other 3 types. Ordinary, nonacute distress that maps onto an existing pattern should continue to be informed by longitudinal pattern and semantic context, and only signals that cross a clinically meaningful threshold should shift the balance toward the present moment. Ambiguous or subthreshold signals should be represented as uncertainty to be monitored. We do not specify universal numeric thresholds because appropriate calibration is implementation-specific and requires clinical oversight. We emphasize that therapeutically informed memory, including state-responsive memory, may improve continuity and safety, but it does not substitute for clinical judgment, diagnosis, the therapeutic relationship, or treatment planning by qualified professionals.

## How the 4 Types Work Together

The value of this framework lies not in the individual types but in the relationships between them. In practice, a given interaction may draw on all four simultaneously: a user’s current distress (state-responsive) may be interpreted in light of a specific recent event (episodic), understood as consistent with a recurring pattern (pattern), and contextualized by stable background information (semantic).

This also means that the framework requires explicit rules for how memory types interact and how conflicts between them are resolved. A past pattern that contradicts a user’s current self-report, or background context that seems inconsistent with recent events, requires a principled approach to weighting. The framework surfaces these tensions as explicit design decisions rather than leaving them to emerge unpredictably from unstructured retrieval. The core principle is that memory use should be context-dependent and function-specific rather than automatic and that the current state retains priority when user safety or acute need is at stake.

**Figure 1.**
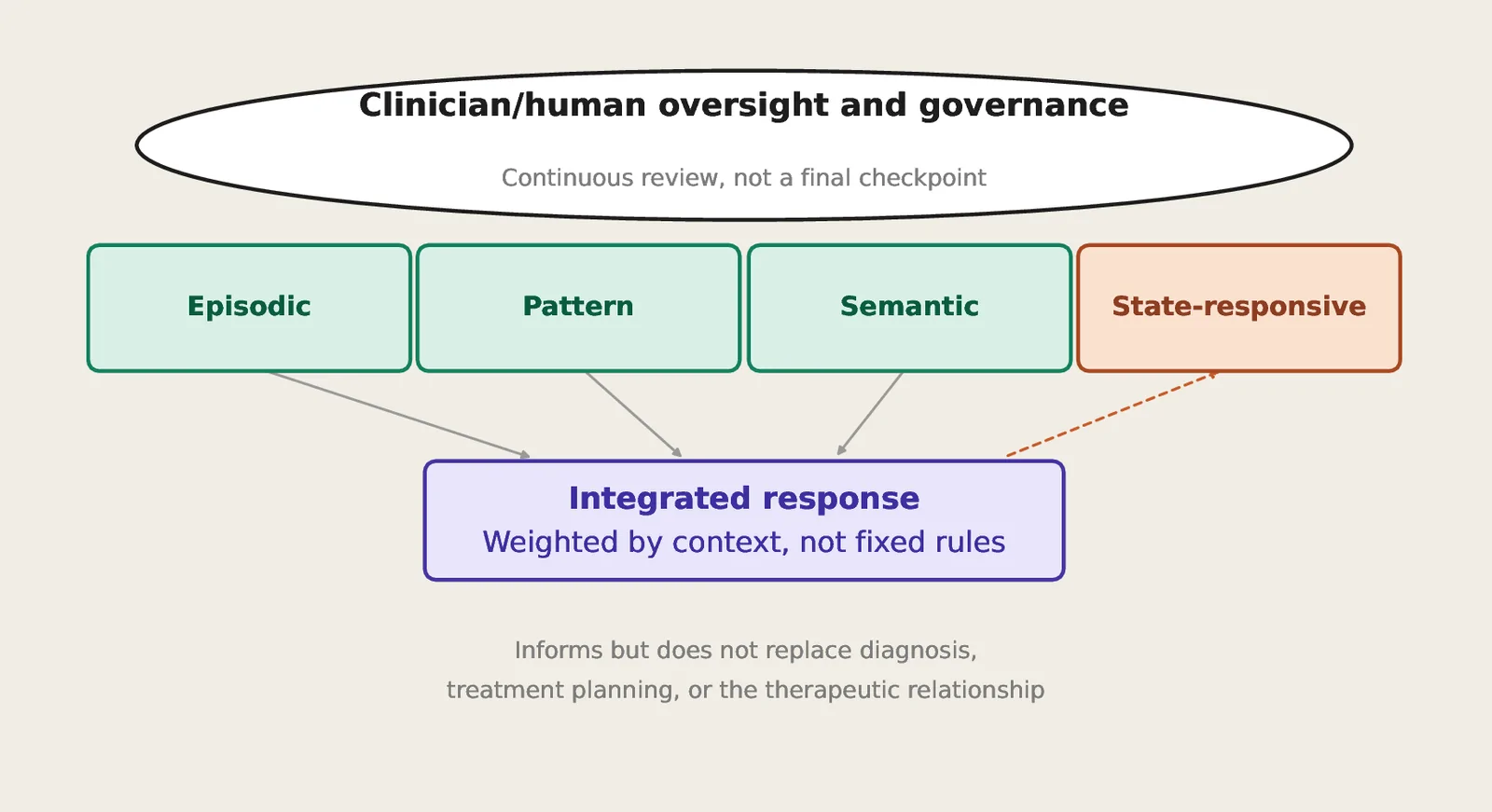
Episodic, pattern, and semantic memory converge into the system’s response to a given interaction, while state-responsive memory can override or take priority when acute risk signals are detected. All output remains subject to clinician oversight and governance.

Figure 1 illustrates how the 4 memory types integrate within a single interaction and how state-responsive memory can take priority when acute risk is detected.

A brief example illustrates how the 4 types combine in practice. Consider a user with an established pattern of withdrawing under caregiver strain (pattern memory), known caregiver status for an ill parent (semantic memory), and a hospitalization she discloses this session (episodic memory). If the user expresses fatigue but no risk indicators, the system draws on all three to recognize the family cycle and check in on her caregiving load. If the user instead expresses hopelessness or intent to harm, state-responsive memory takes over, suspending the pattern-focused interaction and prioritizing crisis support while the other 3 diminish rather than being discarded. This illustrates the framework’s integration logic conceptually; independent reproducible implementation remains an important next step.

Temporal decay and persistence also differ by memory type and require distinct governance. Episodic memory requires timestamps and contextual relevance scoring [17] so that older events are weighted appropriately. Pattern memory requires mechanisms for reinforcement, weakening, retirement, or contradiction handling as new evidence accumulates [16] so that a pattern can lose established status rather than remaining fixed indefinitely. Semantic memory requires periodic confirmation [19] so that background context does not lose value as the user’s circumstances change. State-responsive memory is inherently short-lived. Any retention of state-level information beyond the session it appeared in requires an explicit rationale and dedicated governance [27].

Failure modes are also specific to memory type. Episodic failures involve retrieving irrelevant, misdated, or emotionally harmful past events. Pattern failures involve falsely identifying a recurring pattern, missing a real one, or reinforcing a maladaptive self-narrative by repeatedly surfacing it [21,22]. Semantic failures involve retaining outdated or incorrect background context. State-responsive failures involve failing to detect acute distress, overescalating ordinary distress, or overriding longitudinal context too aggressively [25,26]. A fifth, cross-memory conflict failure occurs when current state, long-term pattern, and semantic context point in different directions and the system lacks a principled way to reconcile them. Table 1 summarizes each memory type with its clinical mapping, representation, main risks, governance needs, and candidate evaluation metrics.

**Table 1.** Summary of the therapeutically informed memory framework.

Memory type	Information captured	Therapeutic/design function	Example	Main risks/failure modes	Governance needs	Candidate evaluation metrics
Episodic	Discrete, time-bound events (arguments, distress episodes, disclosures)	Narrative continuity; situates current concerns within personal history	A stressful week culminating in a specific argument	Retrieving irrelevant, misdated, or emotionally harmful past events	Timestamps; contextual relevance scoring; selective, structured retrieval	Retrieval precision/recall; contextual relevance of retrieved events
Pattern	Recurring cognitive, emotional, and behavioral regularities across sessions	Inferential layer identifying maintaining factors, cognitive distortions, avoidance cycles, schema patterns, relapse signatures	Repeated withdrawal during periods of low mood	False-positive or false-negative pattern identification; reinforcing maladaptive self-narratives	Candidate-to-established thresholds; multisource corroboration; decay/contradiction handling; clinical oversight	False-positive/negative rate; sessions to establishment; rate of retirement after contradictory evidence
Semantic	Stable background context: relationships, living situation, employment, life history	Interpretive frame for episodic and pattern-level information	Primary caregiver status for an ill family member	Retaining outdated or incorrect background information	Structured fields; periodic reconfirmation; distinct from dynamic session data	Accuracy of semantic memory after user correction
State-responsive	Real-time emotional/psychological state; acute risk signals	Real-time risk assessment, crisis response, and safety prioritization	Expressed hopelessness or crisis-level distress	Failing to detect acute distress; overescalating ordinary distress; overriding longitudinal context too aggressively	Session-scoped, nonpersisted by default; graded, clinically supervised thresholds	Activation latency; sensitivity/specificity of acute risk detection

## Illustrative Example: Yuna

Because several authors have professional and financial relationships with Yuna, the following section should be read strictly as an illustrative implementation example. It does not constitute independent evaluation or validation of the proposed framework. The framework described above is a conceptual proposal, and its value depends in part on whether the distinctions it draws are practically implementable in real AI mental health systems. Yuna, a clinician-informed AI-powered digital mental health intervention deployed in workplace settings, illustrates one way the framework for therapeutically informed memory can be operationalized in practice. Yuna is included here only to illustrate how a therapeutically informed memory architecture might be implemented in a real-world platform; evaluation of whether such architecture improves clinical outcomes remains a future empirical question. Preliminary, uncontrolled within-session findings on self-reported mood and stress [28] motivated our interest in this platform as an illustrative case but are not presented as evidence for the framework itself. The following description is intended to ground the framework in a concrete, real-world use case and illustrate how memory architecture may support the longitudinal engagement that preliminary outcome data suggest is clinically relevant.

Yuna’s architecture organizes memory into distinct functional layers that correspond closely to the 4 types described in this framework. Each layer captures different information and serves a different role in how the system interprets and responds to the user across sessions.

Episodic memory in Yuna’s implementation captures discrete, time-bound experiences reported by the user during sessions, including specific events, moments of distress, and contextually significant disclosures. These are stored in a way that preserves their temporal sequence, allowing the system to reference prior experiences when relevant and to situate current concerns within the user’s personal history. This is consistent with the design principle that episodic memory should support narrative continuity rather than simply accumulating undifferentiated conversational history [17].

Pattern memory functions through a process of cross-session aggregation. As a user engages with Yuna over time, the AI system identifies recurring themes in cognition, emotion, and behavior, for example, a consistent tendency to withdraw during periods of stress or a pattern of self-critical thinking following perceived failure. These patterns are not inferred from a single session but emerge through accumulation across a defined progression: from candidate status, where a pattern is first observed, through active reinforcement, to an established pattern once sufficient evidence has accumulated across sessions. New interactions either reinforce or modify the pattern’s status accordingly. This reflects the clinical principle of longitudinal case formulation, wherein recurring patterns are often invisible within any single encounter [5]. In Yuna’s implementation, pattern memory is operationalized through 3 complementary data collection methods: AI inference from conversational content, in-session inquiry prompting users to reflect on their cognitive patterns, and direct user input through structured self-report questions [29]. These patterns are stored in a temporally organized graph structure that connects cognitive, emotional, and behavioral nodes across sessions, enabling the application of identified patterns across multiple interaction contexts.

Semantic memory stores stable background context about the user, including information about their relationships, work environment, and significant life circumstances. This information provides the interpretive frame within which episodic and pattern-level data are understood, and it is maintained separately from more dynamic information to preserve accuracy. When background context changes, for example, a significant life event or a change in employment, the semantic layer is updated accordingly.

State-responsive memory operates at the session level, tracking the user’s current emotional and psychological condition in real time as the interaction unfolds. In situations where the user expresses acute distress or signals that are consistent with elevated risk, this layer takes functional priority over the other three. The AI system’s response is guided primarily by the current state rather than by historical patterns or background context, consistent with the clinical principle that immediate safety and stabilization take precedence over longitudinal understanding in moments of acute need [23,24]. Yuna’s implementation includes crisis detection methods intended to activate under these conditions, suspending standard coaching and directing the user toward crisis support resources.

The interaction between these 4 layers reflects the framework’s core principle: memory use should be context-dependent rather than automatic. In a typical session, Yuna’s AI system draws on all four simultaneously: current state informs the immediate response, episodic history provides situational context, pattern memory shapes the interpretation of recurring concerns, and semantic memory grounds everything in the user’s stable background. In sessions where no significant distress is present, the balance shifts toward longitudinal understanding. In sessions where distress signals are prominent, state-responsive memory takes precedence, and the other layers recede.

Several design features of Yuna’s implementation illustrate the practical tensions the framework identifies. The question of how much historical context to surface in a given session, how to weight a pattern that appears to be shifting against one that has been stable, and how to determine when a current state signal is sufficient to override longitudinal guidance are all decisions that require explicit design logic rather than general retrieval. Yuna’s clinician-informed protocols provide one approach to these decisions, though the underlying principles are generalizable beyond any single platform. This description emphasizes design logic rather than product performance and should not be read as a claim that Yuna’s specific implementation is optimal or superior to alternative approaches.

It is important to note that this description is based on Yuna’s design as an illustrative case and should not be interpreted as evidence of clinical efficacy. The framework itself does not depend on Yuna’s specific implementation; rather, Yuna demonstrates that the conceptual distinctions the framework proposes are practically addressable in a real system. Whether this approach to memory organization produces meaningfully better clinical outcomes than alternatives remains an open and important empirical question.

## Design Implications

The framework proposed in this paper has several practical implications for how AI-powered mental health systems are designed, evaluated, and refined. These implications extend beyond Yuna as a specific platform and speak to the broader challenge of developing AI tools that can support users meaningfully across time.

The first and most fundamental implication is that memory should be treated as a core system function, not an ancillary feature added to improve conversational feel. Current AI-powered mental health systems are predominantly evaluated on the quality of individual interactions, including response appropriateness, user satisfaction, and short-term symptom change [8,9]. These are important measures, but they do not capture whether the system is building and using longitudinal understanding in clinically meaningful ways. Evaluation frameworks need to expand to include assessment of how well systems track change over time, recognize recurring patterns, and adapt their responses accordingly. This requires longitudinal study designs with repeated validated outcome measures, which remain underrepresented in the current literature on AI mental health tools [25,30].

Second, the framework highlights that different memory types require different data infrastructure. Episodic memory requires temporally organized storage with selective retrieval mechanisms. Pattern memory requires cross-session aggregation and pattern detection capabilities. Semantic memory requires structured, high-accuracy storage that is maintained separately from dynamic session data. State-responsive memory requires real-time updating with prioritization logic that can override other memory types under specific conditions. These are not trivial engineering requirements, and conflating them into a single undifferentiated memory store, as most current systems do, produces predictable design failures. Development teams need to distinguish between these requirements from the outset rather than treating memory as a feature to be layered onto an existing conversational architecture.

Third, the framework raises important questions about transparency and user awareness. Users interacting with an AI-powered mental health system over multiple sessions may reasonably want to understand what the system remembers, how it uses that information, and when and why it prioritizes certain types of information over others [27]. This is especially salient when state-responsive memory overrides longitudinal context, as users in distress may experience a noticeable shift in the system’s behavior without understanding why. Plain-language explanations of how memory functions in these systems are not merely a transparency courtesy; they are a component of informed engagement and trust [27]. Developers should consider how memory architecture is communicated to users and whether users have meaningful agency over what is retained and how it is applied. This should include the ability to view what the system remembers, correct inaccurate memories, delete specific memories, reset pattern memory, pause memory collection, understand the distinction between personalization memory and therapeutic memory, request deletion of risk-related or sensitive memories, understand why a particular memory was retrieved in a given moment, and consent separately to different memory types [27]. These are presented as design requirements implied by the framework rather than as features already implemented in any specific system, including Yuna.

Fourth, the role of clinician involvement in memory design deserves attention. The memory types proposed in this framework are grounded in clinical concepts, including case formulation, measurement-based care, and crisis-informed practice. Translating these concepts into functional system design requires ongoing collaboration between clinicians and engineers, not a one-time consultation at the point of development. As patterns are detected, as semantic context is updated, and as state-responsive memory is triggered, clinical judgment about what constitutes a meaningful signal, a genuine pattern, or a genuine risk indicator needs to be embedded in the system’s logic and reviewed over time [2]. AI systems that lack this ongoing clinical oversight risk encoding clinically inappropriate thresholds or failing to detect signals that a trained clinician would recognize. We also reiterate that therapeutically informed memory may improve continuity and safety, but it does not substitute for clinical judgment, diagnosis, the therapeutic relationship, or treatment planning by qualified professionals.

Fifth, the framework should not be read as imposing identical requirements on every system it might describe. Memory requirements differ depending on whether a given system is a wellness or coaching tool, a workplace mental health support tool such as Yuna, a digital therapeutic, a clinician-supervised intervention, a crisis-support adjunct, or an AI system embedded in formal care [2]. The rigor required for pattern validation, the specificity of state-responsive thresholds, and the governance surrounding retention and deletion should scale with the level of clinical risk and clinical integration involved. A wellness chatbot and a clinician-supervised digital therapeutic are not held to the same standard, even though both may benefit from the same conceptual organization of memory.

Sixth, current evaluations of AI-powered mental health systems assess user satisfaction, symptom change, and engagement but do not directly assess whether the system’s use of longitudinal information improved the quality of its responses or the relevance of its clinical support [26]. Developing metrics for memory quality, including measures of pattern recognition accuracy, contextual relevance of retrieved episodic information, and appropriate prioritization of state-responsive signals, would represent a meaningful methodological advance for the field. Preliminary candidate metrics organized by memory type, including retrieval precision/recall and contextual relevance for episodic memory, false-positive/false-negative rates and time-to-establishment for pattern memory, and rate of successful pattern retirement or decay after contradictory evidence, postcorrection accuracy for semantic memory, and activation latency and sensitivity/specificity of acute risk detection for state-responsive memory, with user-rated trust, perceived control, and helpfulness versus intrusiveness, are summarized in Table 1. Finally, the framework points toward the importance of evaluating what happens when memory systems fail. Overretrieval of past context, misidentification of patterns, failure to update semantic information when circumstances change, and delayed activation of state-responsive priority are all failure modes with real clinical consequences. Prospective evaluation of AI-powered mental health systems should include systematic assessment of these alongside assessment of positive outcomes, consistent with calls in the broader AI safety literature for adversarial testing and red teaming (systems are deliberately probed for failure and unsafe features) of AI-powered mental health tools [2].

## Limitations

This paper has several limitations. The framework is conceptual and has not been empirically validated. Whether organizing memory in this way produces measurably better outcomes than existing approaches has not been tested. The illustrative example is drawn from a single platform, and it has not been independently tested or evaluated. It remains unclear how well the framework generalizes across different AI architectures, model types, and deployment contexts beyond Yuna, and its performance implications are unknown across diverse user populations and levels of clinical risk. Validation, including empirical methods such as expert interviews with psychotherapists and professional coaches, is an important and necessary next step. This paper is intended to organize the field’s thinking and generate testable hypotheses.

## Conclusion

Memory in AI-powered mental health systems is currently underconceptualized. Most systems treat it as a single mechanism for personalization, without distinguishing between types of information that serve fundamentally different therapeutically relevant functions. The framework proposed here organizes memory into four functionally distinct types: episodic, pattern, semantic, and state-responsive. Together, they provide a structured approach to how AI mental health systems can build and use longitudinal understanding in ways that more closely reflect how memory functions in effective clinical care.

The application of this framework to Yuna illustrates that these distinctions are practically addressable in a real system, though prospective controlled research is needed before causal conclusions about outcomes can be drawn [28]. It is intended as a generalizable conceptual contribution, not a product-specific claim.

Open questions remain. Validated metrics for memory quality do not yet exist. The conditions under which longitudinal memory use improves outcomes relative to single-session approaches have not been empirically established. The ethical dimensions of persistent memory in AI mental health systems require dedicated attention as these tools scale [27].

Systems that lack a principled approach to memory may provide adequate single-session support while failing to deliver the longitudinal understanding that distinguishes meaningful mental health support from personalization alone. Addressing this gap is not primarily a technical challenge. It is a conceptual and clinical one.
